# Evaluating the Effects of Rewards and Schedule Length on Response Rates to Ecological Momentary Assessment Surveys: Randomized Controlled Trials

**DOI:** 10.2196/45764

**Published:** 2023-10-19

**Authors:** Sarah Edney, Claire Marie Goh, Xin Hui Chua, Alicia Low, Janelle Chia, Daphne S Koek, Karen Cheong, Rob van Dam, Chuen Seng Tan, Falk Müller-Riemenschneider

**Affiliations:** 1 Physical Activity and Nutrition Determinants in Asia Programme Saw Swee Hock School of Public Health National University of Singapore and National University Health System Singapore Singapore; 2 Singapore Health Promotion Board Singapore Government Singapore Singapore; 3 Department of Exercise and Nutrition Sciences and Epidemiology Milken Institute of Public Health The George Washington University Washington DC, VA United States; 4 Saw Swee Hock School of Public Health National University of Singapore and National University Health System Singapore Singapore; 5 Yong Loo Lin School of Medicine National University of Singapore Singapore Singapore; 6 Digital Health Center Berlin Institute of Health Charité-Universitätsmedizin Berlin Berlin Germany

**Keywords:** experience sampling, ambulatory assessment, compliance, mobile phone

## Abstract

**Background:**

Ecological momentary assessments (EMAs) are short, repeated surveys designed to collect information on experiences in real-time, real-life contexts. Embedding periodic bursts of EMAs within cohort studies enables the study of experiences on multiple timescales and could greatly enhance the accuracy of self-reported information. However, the burden on participants may be high and should be minimized to optimize EMA response rates.

**Objective:**

We aimed to evaluate the effects of study design features on EMA response rates.

**Methods:**

Embedded within an ongoing cohort study (Health@NUS), 3 bursts of EMAs were implemented over a 7-month period (April to October 2021). The response rate (percentage of completed EMA surveys from all sent EMA surveys; 30-42 individual EMA surveys sent/burst) for each burst was examined. Following a low response rate in burst 1, changes were made to the subsequent implementation strategy (SMS text message announcements instead of emails). In addition, 2 consecutive randomized controlled trials were conducted to evaluate the efficacy of 4 different reward structures (with fixed and bonus components) and 2 different schedule lengths (7 or 14 d) on changes to the EMA response rate. Analyses were conducted from 2021 to 2022 using ANOVA and analysis of covariance to examine group differences and mixed models to assess changes across all 3 bursts.

**Results:**

Participants (N=384) were university students (n=232, 60.4% female; mean age 23, SD 1.3 y) in Singapore. Changing the reward structure did not significantly change the response rate (*F*_3,380_=1.75; *P*=.16). Changing the schedule length did significantly change the response rate (*F*_1,382_=6.23; *P*=.01); the response rate was higher for the longer schedule (14 d; mean 48.34%, SD 33.17%) than the shorter schedule (7 d; mean 38.52%, SD 33.44%). The average response rate was higher in burst 2 and burst 3 (mean 50.56, SD 33.61 and mean 48.34, SD 33.17, respectively) than in burst 1 (mean 25.78, SD 30.12), and the difference was statistically significant (*F*_2,766_=93.83; *P*<.001).

**Conclusions:**

Small changes to the implementation strategy (SMS text messages instead of emails) may have contributed to increasing the response rate over time. Changing the available rewards did not lead to a significant difference in the response rate, whereas changing the schedule length did lead to a significant difference in the response rate. Our study provides novel insights on how to implement EMA surveys in ongoing cohort studies. This knowledge is essential for conducting high-quality studies using EMA surveys.

**Trial Registration:**

ClinicalTrials.gov NCT05154227; https://clinicaltrials.gov/ct2/show/NCT05154227

## Introduction

Ecological momentary assessment (EMA) surveys are a method of capturing self-reported information on experiences in real-time, real-life settings. In EMA studies, participants are prompted to respond to brief sets of questions, often multiple times per day for several days [1]. Although EMA has been used for decades [2], this approach has recently increased in popularity as evidenced by the number of recent review studies [3-15]. These reviews have examined the use of EMAs to study various health-related behaviors and experiences, including stress [4], mood and anxiety disorders [10], social interactions [5], physical activity, eating behaviors, tobacco smoking, sexual health, and alcohol consumption [6]. Other reviews have covered the applications of EMA within clinical psychology [3,9,12,15] and methodological considerations [7,11].

There are 3 key advantages of using EMA over traditional retrospective surveys. First, the recall period is short, typically covering current or very recent experience, thus minimizing recall biases [16,17]. Second, ecological validity is enhanced as experiences are reported in the context of daily life. Third, EMA can be delivered on intensive and repeated schedules to capture patterns and dynamic interactions between experiences that may occur as frequently as weekly, daily, hourly, or more [18]. These advantages are further enhanced by technological developments that have made it possible to deliver EMA surveys via a smartphone [19] rather than via pen and paper assessments.

Integrated into longitudinal cohort studies, periodic *bursts* of EMA surveys (ie, repeated rounds of EMA surveys [20,21]) could advance our understanding of trajectories of health and health-related behaviors [18,22]. Such an approach overcomes the methodological limitations of traditional cohort studies, where assessments are repeated only months, years, or decades apart, by capturing within-person variations in health and health-related behaviors and dynamic interactions between them and contextual determinants in real-life settings. A few such studies are ongoing in the United States [23-25]. One study currently underway at the National University of Singapore (NUS) is Health@NUS, which aims to examine the health and health-related behaviors of approximately 1000 university students (ClinicalTrials.gov NCT05154227).

Nonresponse to EMA surveys may erode the advantages of this approach. EMA protocols must balance comprehensive coverage of the constructs of interest (eg, health-related behaviors such as physical activity or eating and experiences such as stress or mood) against acceptable participant burden [26,27]. Questions contained within EMA surveys must accurately assess each construct and be implemented within a sampling strategy that matches the expected occurrence (or fluctuation) of the construct in daily life [20,28]. However, if each EMA survey has many questions or is sent frequently, respondents may find the EMAs intrusive or difficult to respond to in the context of daily life. Placing high burden on respondents may result in nonresponse to EMA surveys and an incomplete picture of the construct of interest [29,30].

Currently, little is known about maximizing data completeness in EMA studies. Some studies have found that missing data are related to participant characteristics such as age or personality traits [31] and to study design factors such as the incentives offered to participants, the number of days of monitoring, or the number of surveys per day or questions per survey [8,11,32,33]. Similarly, the content or complexity of the included questions may influence participants’ ability or willingness to respond. If such factors are related to missing data, then they require careful consideration when designing EMA studies. This is particularly important within longitudinal studies that implement bursts of EMA alongside other study requirements (eg, health screenings, continuous digital assessments, biometric assessments, and traditional questionnaires), as the burden on participants may be considerable and willingness to engage with the study requirements may decrease over time. In addition to concerns about missing EMA data, undue burden may result in poorer quality of data (eg, owing to careless responding to EMA surveys [33]), to other study requirements being missed, or, in the worst case, withdrawal from the study.

Furthermore, studies that implement repeated bursts of EMA surveys face unique challenges when compared with *one-off* or *single-burst* EMA studies. The start date of single-burst studies is often clear—a prespecified date or directly following enrollment into a study or contact with the research team. Conversely, when multiple bursts of EMAs are implemented, the start date may be clear for the first burst of EMA if it coincides with the recruitment date. However, the start date of subsequent bursts may only be communicated electronically (eg, an email or push notification) or not at all (eg, participants just receive the first EMA survey), and this may have important implications for response rates to the upcoming round. If the communication strategy is not optimum, it follows that participants may not be able to respond to all EMA surveys in all EMA bursts.

This study aims to evaluate progressive changes made to the implementation strategy for bursts of EMA surveys embedded within an ongoing cohort study. Our objectives were as follows:

1. *Aim 1*: to evaluate whether offering different reward structures for completing EMA surveys would lead to an increase in the response rate relative to the control group.
2. *Aim 2*: to evaluate whether implementing a 7-day EMA schedule (intervention group) would improve the response rate relative to a 14-day schedule (control group).
3. *Secondary aim—aim 3*: To compare the overall response rates across 3 bursts of EMA surveys following changes to the EMA implementation protocol.

## Methods

This study evaluated participants’ response rates to the first 3 bursts of EMA surveys nested within an ongoing prospective cohort study, Health@NUS (ClinicalTrials.gov NCT05154227).

### Health@NUS

Full details of Health@NUS are available elsewhere [34]. Briefly, Health@NUS uses traditional and digital strategies to capture health-related behaviors and related factors over a 2-year period as students complete their university education and as many of them transition into postuniversity work and life. Throughout the 2-year study, participants repeat traditional questionnaires and biometric assessments (baseline and 1- and 2-y follow-up). Movement behaviors (ie, physical activity, sedentary behavior, and sleep [35]) were monitored continuously using a wearable device (Fitbit Versa Lite), and a smartphone app tracked dietary intake and delivered up to 5 bursts of EMA surveys per year. These EMA data extend and contextualize the information from the traditional and digital assessments with questions covering movement (sleep, physical activity, and screen time) and diet (whether food was eaten, where it was eaten, what was eaten, activities while eating, and satiety after eating) and stress, fatigue, and mood. By combining bursts of EMA surveys with other digital technologies, Health@NUS will capture and describe the temporality of experiences within and between days, and their effects on health over time, at a level of granularity not previously possible.

The schedule for the first burst of Health@NUS EMA surveys was designed based on the available literature [11,32], the need to capture multiple constructs as succinctly as possible, and our experiences implementing EMA in the local context [22,36,37]. Despite this approach, the response rate to the first burst was low. On average, participants completed only 26% of the surveys they received, well below the 70% or higher response rate reported in other studies with comparable populations [11,32,38] and the recommended acceptable threshold of 80% [2,39]. This low response rate prompted the study team to carefully review the EMA implementation strategies and schedule with the overall aim to increase the response rate in future bursts.

### This Study

**Figure 1:**
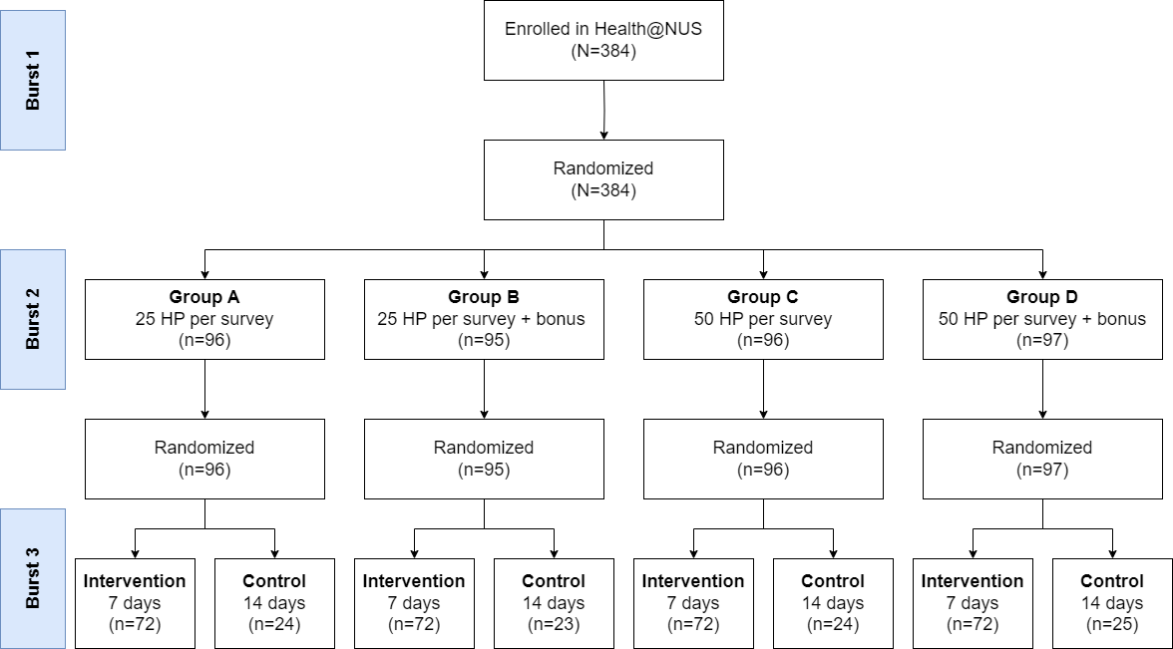
Flow of participants through the nested randomized controlled trials. HP: Health Points.

To evaluate these changes, we implemented 2 randomized controlled trials (RCTs) within the ongoing Health@NUS study. The practical nature of these nested RCTs (ie, to act to improve overall EMA survey response rate in an ongoing study) meant that publishing a protocol before starting the study was not feasible. The outcome variable (response rate) was specified *a priori*. The flow of participants through these RCTs is shown in Figure 1*.*

### Participants

Participants were recruited to Health@NUS via email, campus posters, and word of mouth. To be eligible, participants had to (1) be a full-time student at the NUS, (2) be aged 18 to 26 years, (3) be a citizen or permanent resident of Singapore, and (4) own a smartphone compatible with the study app (ie, minimum iOS 10 or Android 7). Recruitment was ongoing at the time of writing this paper.

There were no additional eligibility criteria for this study. A total of 384 students who joined Health@NUS during the first wave of recruitment between October 2020 and March 2021 were included. During study enrollment, participants provided written informed consent to receive short surveys (EMAs, <10 min each) via the study app (HiSG app). Participants were advised that the EMA surveys were optional but highly encouraged and that they would be compensated for answering them. No specific details of the survey timing, frequency, or the compensation were provided during the consent process.

### EMA Details

#### Overview

This study was based on the first 3 bursts of EMA surveys, delivered on the following dates:

1. *Burst 1*: April 24 to May 7, 2021 (baseline)
2. *Burst 2*: July 19 to August 1, 2021 (aim 1, reward RCT)
3. *Burst 3*: October 11 to October 24, 2021 (aim 2, schedule length RCT)

The EMA questions asked about movement behaviors, eating behaviors, the context of these behaviors, and emotional states. The content and number of questions in each survey varied (minimum: 1 question; maximum: 12 questions; Multimedia Appendices 1 and 2). As this study focused on the overall response rate, the details of the content of the EMA survey questions are not described here.

In each burst, up to 6 EMA surveys were delivered per day on a time-stratified sampling schedule [39]. EMA surveys were scheduled to be sent at a random time within the following fixed time windows: 8:30 AM to 9:30 AM (survey 1), 11 AM to noon (survey 2), 1:30 PM to 2:30 PM (survey 3), 4 PM to 5 PM (survey 4), 6:30 PM to 7:30 PM (survey 5), and 9 PM to 10 PM (survey 6).

In all 3 bursts of EMA surveys, participants were notified of each EMA survey via a push notification, plus a second push notification sent 25 minutes later if the EMA survey had not already been answered. Participants had 45 minutes to respond to each EMA survey, starting from the time of the first push notification.

#### Burst 1 of EMA Surveys

**Figure 2:**
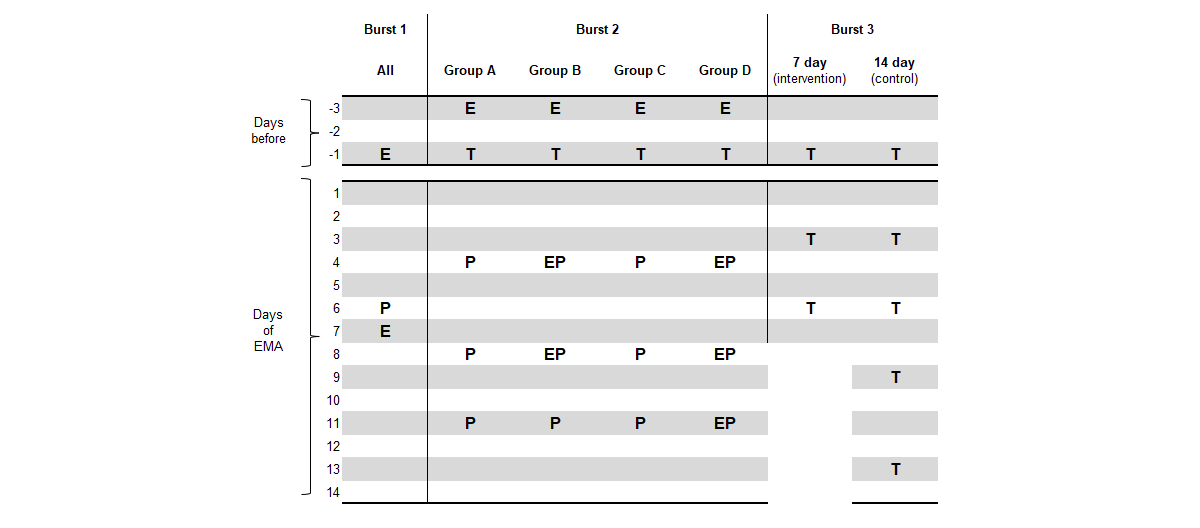
Overview of general reminders sent before and during each ecological momentary assessment (EMA) burst. Reminder sent via email (E), text message (T), or push notification (P). Group A: 25 Health Points (HP) per completed EMA survey. Group B: 25 HP per completed EMA survey + bonus HP available. Group C: 50 HP per completed EMA survey. Group D: 50 HP per completed EMA survey + bonus HP available. An additional 2 push notifications are sent per EMA survey (first push notification, second push notification 25 min later, if survey remains unanswered).FIG2OV~1.PNGd.

Before burst 1 (baseline), the participants received an email to notify them of the upcoming EMA burst. A second reminder email and one push notification reminder were sent midway through the EMA burst (see the left-hand side of Figure 2).

**Figure 3:**
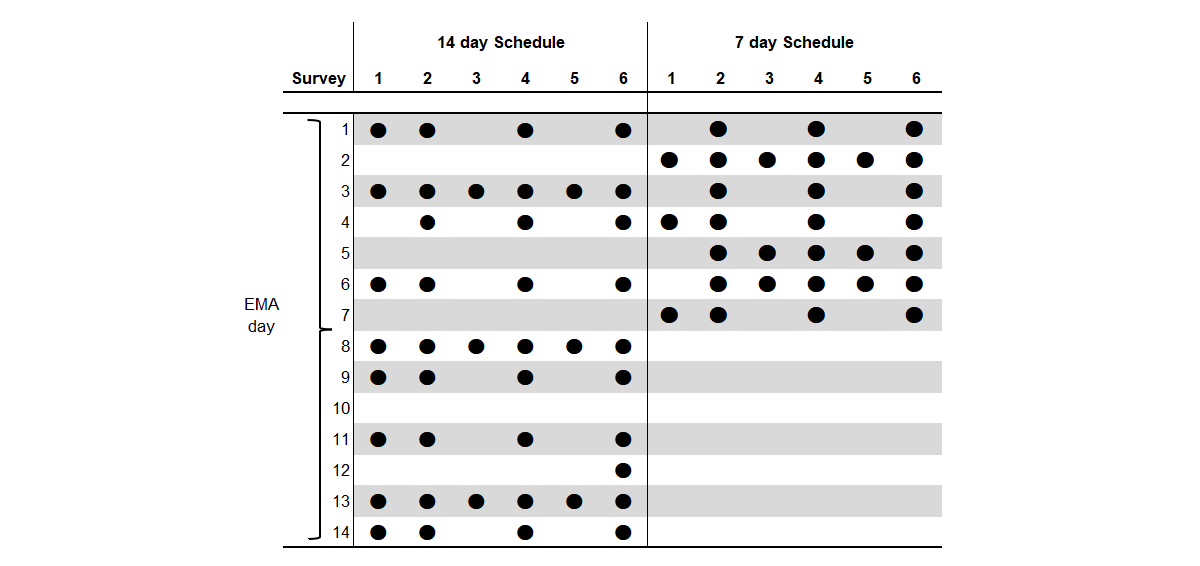
Overview of the 14- and 7-day ecological momentary assessment (EMA) schedules. The black dot indicates that an EMA survey was sent on this day in this time window. Surveys were sent at random times within the following time windows: survey 1 (8:30-9:30 AM), survey 2 (11 AM to noon), survey 3 (1:30-2:30 PM), survey 4 (4-5 PM), survey 5 (6:30-7:30 PM), survey 6 (9-10 PM). Burst 1 and 2: all participants (N=384) received the 14-day EMA schedule (burst 1: 42 EMA surveys and burst 2: 41 EMA surveys as survey 6 on day 9 was not sent due to a technical glitch). Burst 3: intervention group participants (n=288) received the 7-day schedule (30 EMA surveys), and control group participants (n=96) received the 14-day schedule (42 EMA surveys).

During burst 1, all participants received 42 EMA surveys over 10 days within a 14-day period (see the left-hand side of Figure 3). Participants received 25 Health Points (HP) for completing each individual EMA survey, up to a total of 1050 HP (equivalent to approximately SG $7 [US $5.1]). In Singapore, HP can be earned by participating in a range of health promotion programs such as yearly physical activity interventions [40]. HP are accumulated in one central e-wallet and can be exchanged for vouchers.

#### Burst 2 of EMA Surveys

The EMA schedule was identical to the 14-day schedule implemented in burst 1 (see the left-hand side of Figure 3) with the exception that survey 6 on day 9 was not sent because of a technical glitch in the study app, resulting in 41 surveys being sent in total.

Before the start of burst 2, participants received an email and an SMS text message to notify them of the upcoming EMA burst. In addition, all participants received a push notification on their smartphone every 3 days to remind them to complete the EMA surveys (Figure 2) and participants in groups B and D (Table 1) also received an email reminder every 3 days.

In burst 2 of EMA surveys, there were 4 different reward structures provided for completing EMA surveys: a control group that received 25 HP per completed EMA survey (group A), a group that received 25 HP per completed EMA survey plus bonus HP for completing >50% or 80% of EMA surveys (group B, ie, 50% or 80%=completing >20 or >32 EMA surveys, respectively), a group that received 50 HP per completed EMA survey (group C), and a group that received 50 HP per EMA survey plus bonus HP for completing >50% or 80% of EMA surveys (group D; Table 1).

To evaluate whether changing the reward structure led to an increase in the EMA response rate (*aim 1*, reward RCT), participants were randomly allocated on a 1:1:1:1 ratio to group A, B, C, or D before burst 2. An independent allocation officer used a random number generator to determine the allocation sequence. The sequence was integrated into the HiSG app and participants were automatically assigned to their respective group for burst 2 of the EMA surveys.

The participants were not explicitly informed of their group allocation or of the existence of different reward groups. However, participants were notified that rewards were available and participants in group B and group D were notified of the bonus HP available and of the completion rate threshold that they needed to meet to receive them (ie, at least 50% or 80% completion) via the reminder emails and push notifications (Figure 2).

**Table 1:** Reward structure for the 4 intervention groups^a^.

			Group A		Group B		Group C		Group D
HP^b^/survey			25		25		50		50
**Bonus HP^c^**									
	>50% EMA^d^ surveys completed	N/A^e^		500		N/A		500	
	>80% EMA surveys completed	N/A		1000		N/A		1000	
**Total possible reward**									
	HP	1050		2050		2100		3100	
	Approximate value, SG $ (US $)	7.0 (5.1)		13.7 (10.0)		14.0 (10.3)		20.7 (15.2)	

^a^Rewards were provided as Health Points.

^b^HP: Health Points.

^c^Participants could receive either the 50% or 80% completion bonus, not both.

^d^EMA: ecological momentary assessment.

^e^N/A: not applicable. Participants in these groups were not eligible for bonus HP.

#### Burst 3 of EMA Surveys

The original 14-day schedule was condensed into 7 days, and the overall number of EMA surveys was reduced to 30 (see the right-hand side of Figure 3). Early morning surveys (survey 1, 8:30-9:30 AM) were removed from the 7-day schedule where possible as these received the lowest response rate in previous bursts. The control group received the original 14-day schedule (identical to the 14-d schedule used in bursts 1 and 2; see the left-hand side of Figure 3). The reward structure was reverted to that of burst 1 (ie, 25 HP/completed survey for all participants) as preliminary analyses of the reward RCT (ie, *aim 1*) data indicated no significant between-group difference in the response rate to EMA surveys.

Before the start of burst 3, participants received an SMS text message (instead of an email) notifying them of the upcoming EMA burst plus they received an SMS text message every 3 days to remind them to complete the EMA surveys (total number of SMS text messages sent/participant: 5 for those receiving a 14-d schedule and 3 for those receiving a 7-d schedule [Figure 2]). Participants were not directly informed of their group allocation (14 or 7 d).

The second nested RCT aimed to evaluate whether a condensed EMA schedule would achieve a higher response rate (hereafter referred to as *schedule length RCT*). We hypothesized that the condensed 7-day schedule (*intervention*) would achieve a higher response rate than the original 14-day schedule (*control*) because some studies have reported declining response rates over time [41-43]. As such, we conducted a 2-arm trial in which we randomly allocated participants on a 1:3 allocation ratio (control: intervention). Randomization was stratified by reward RCT groups to ensure that the 1:3 allocation ratio was equal across the 4 reward groups and to ensure that prior reward experience would not have an impact on the results. As before, an independent allocation officer used a random number generator to determine the allocation sequence, and this was integrated into the HiSG app so that participants were automatically assigned to their respective group for burst 3.

### Measures

At baseline, participants self-reported their age (date of birth); sex (male or female); ethnicity (Chinese, Indian, Malay, or Other); marital status (single or never married, currently married, separated but not divorced, divorced, widowed, or refuse to answer); monthly household income (<SG $2000 [US $1466], SG $2000-SG $3999 [US $1466-US $2932], SG $4000-SG $5999 [US $2933-US $4398], SG $6000-SG $9999 [US $4399-US $7331], >SG $10,000 [>US $7332]), refuse to answer, or do not know); whether they are an undergraduate or postgraduate student; and the faculty they are studying in.

Biometric assessments (height in cm and weight in kg) were taken by trained study personnel. BMI was calculated from height and weight measurements and classification recommendations for Asian populations [44] were followed (<18.4 kg/m^2^=underweight; 18.5-22.9 kg/m^2^=normal; 23-27.4 kg/m^2^=overweight; and >27.5 kg/m^2^=obese).

EMA surveys were administered via the HiSG app, and responses were automatically captured by the app and uploaded to a study server in real time.

### Primary Outcome

The primary outcome measure was the response rate (ie, percentage of completed EMA surveys from all sent EMA surveys) for each burst of EMA surveys.

### Statistical Methods

Baseline characteristics were analyzed descriptively. Separate 1-way ANOVAs were used to estimate the effect of changing the reward structure (aim 1, reward RCT) or the schedule length (aim 2, schedule length RCT) on the response rate at burst 2 and burst 3, respectively. A sensitivity analysis was conducted using analysis of covariance to adjust for the response rate at burst 1 (ie, baseline).

Secondary aim 3 was first analyzed using linear mixed model analysis with restricted maximum likelihood estimation to compare the overall response rate across the 3 bursts of EMA surveys following changes to the EMA implementation protocol (illustrated in Figure 2). The model was adjusted for group allocation at burst 2 and burst 3. Pairwise comparisons, with Bonferroni correction for multiple comparisons, were conducted to identify which bursts had significantly different response rates. We also conducted further subgroup analysis for secondary aim 3 with participants who were allocated to the control group for each EMA burst. The subgroup for this analysis was specified as all participants from burst 1 (N=384), data from group-A (control group) participants at burst 2 (n=96), and data from participants in the 4 groups that received the 14-day schedule (control group) at burst 3 (n=96; 24 of these participants also contributed data in burst 2). All analyses were conducted in R software (version 4.1; R Foundation for Statistical Computing).

### Ethical Considerations

Ethics approval was obtained from the National Healthcare Group in Singapore (reference 2019/00285). All participants provided written informed consent before commencing the study and consented for their deidentified data to be used for research purposes. For this study, the maximum compensation available to participants ranged from SG $21 (US $15) to SG $34.66 (US $25) depending on group allocation during the reward RCT and on the number of EMA surveys they completed.

## Results

### Participant Flow

Between October 2020 and March 2021, 384 participants were recruited and enrolled into this study, and data collection was complete by October 2021.

Following burst 1 and before burst 2, the participants were randomized to group A (n=96), group B (n=95), group C (n=96), or group D (n=97). Following burst 2 and before burst 3, participants were randomized to the intervention (7-d EMA schedule, n=288) or control (14-d EMA schedule, n=96) group. Figure 1 presents the details of participant flow through the trial.

### Participant Characteristics

The study participants were predominantly undergraduate students (376/384, 97.9%), female (232/384, 60.4%), of Chinese ethnicity (366/384, 95.3%), and reported as being single or never married (382/384, 99.5%). The mean age of the participants was 23.37 (SD 1.25) years, and most of the participants had a BMI that was classified as either normal weight (203/384, 52.9%) or moderately overweight (114/384, 29.7%). Details of the participant characteristics at baseline for all participants and for each group allocation in the 2 RCTs are presented in Table 2.

For the reward RCT, group A had a slightly higher proportion of participants with a monthly household income of <SG $2000 (<US $1466; compared with the other 3 reward groups). For the schedule length RCT, the 7-day schedule group had a slightly higher proportion of male participants and of participants with a reported monthly household income of <SG $2000 (<US $1466) as compared with the 14-day schedule group. The 7-day schedule group also had fewer participants who reported a BMI in the healthy range as compared with the 14-day schedule group (Table 2).

**Table 2:** Participant characteristics.

		Overall (N=384)	Reward RCT^a^					Schedule length RCT	
			Group A^b^ (n=96)	Group B^c^ (n=95)	Group C^d^ (n=96)	Group D^e^ (n=97)	7-d schedule (n=288)		14-d schedule (n=96)
Age (y), mean (SD)		23.4 (1.3)	23.4 (1.2)	23.6 (1.3)	23.2 (1.1)	23.3 (1.4)	23.4 (1.2)		23.3 (1.4)
**Sex, n (%)**									
	Male	152 (39.6)	36 (37.5)	43 (45.3)	40 (41.7)	33 (34)	120 (41.7)		32 (33.3)
	Female	232 (60.4)	60 (62.5)	52 (54.7)	56 (58.3)	64 (66)	168 (58.3)		64 (66.7)
**Ethnicity, n (%)**									
	Chinese	366 (95.3)	92 (95.8)	91 (95.8)	91 (94.8)	92 (94.8)	277 (96.2)		89 (92.7)
	Other	18 (4.7)	4 (4.2)	4 (4.2)	5 (5.2)	5 (5.2)	11 (3.8)		7 (7.3)
BMI (kg/m^2^), mean (SD)		22.03 (3.23)	22.07 (2.84)	22.26 (3.31)	21.53 (2.95)	22.28 (3.73)	22.15 (3.3)		21.70 (2.96)
**BMI categories (kg/m^2^), n (%)**									
	Underweight: <18.5	47 (12.2)	13 (13.5)	7 (7.4)	15 (15.6)	12 (12.4)	37 (12.8)		10 (10.4)
	Normal: 18.5-<23	203 (52.9)	49 (51)	51 (53.7)	54 (56.3)	49 (50.5)	143 (49.6)		60 (62.5)
	Overweight: 23-<27.5	114 (29.7)	31 (32.3)	31 (32.6)	24 (25)	28 (28.9)	90 (31.3)		24 (25)
	Obese: ≥27.5	20 (5.2)	3 (3.1)	6 (6.3)	3 (3.1)	8 (8.2)	18 (6.3)		2 (2.1)
**Faculty, n (%)**									
	Science and Medicine	155 (40.4)	29 (30.2)	46 (48.4)	40 (41.7)	40 (41.2)	112 (38.9)		43 (44.8)
	Engineering and Computing	86 (22.4)	33 (34.4)	21 (22.1)	13 (13.5)	19 (19.6)	68 (23.6)		18 (18.8)
	Arts and Social Sciences	56 (14.6)	19 (19.8)	7 (7.4)	15 (15.6)	15 (15.5)	44 (15.3)		12 (12.5)
	Business, Accounting and Law	42 (10.9)	6 (6.3)	11 (11.6)	12 (12.5)	13 (13.4)	32 (11.1)		10 (10.4)
	Design and Environment	32 (8.3)	5 (5.2)	8 (8.4)	14 (14.6)	5 (5.2)	23 (8)		9 (9.4)
	Others	13 (3.4)	4 (4.2)	2 (2.1)	2 (2.1)	5 (5.2)	9 (3.1)		4 (4.2)
**Monthly household income (SG $ [US $]), n (%)**									
	<2000 (<1466)	37 (9.6)	13 (13.5)	5 (5)	8 (8.3)	11 (11.3)	31 (10.8)		6 (6.3)
	2000-5999 (1466-4397)	104 (27.1)	22 (22.9)	26 (27.4)	26 (27.1)	30 (30.9)	75 (26)		29 (30.2)
	6000-9999 (4398-7329)	69 (18)	19 (19.8)	15 (15.8)	22 (22.9)	13 (13.4)	52 (18.1)		17 (17.7)
	>10,000 (>7330)	70 (18.2)	21 (21.9)	17 (17.9)	16 (16.7)	16 (16.5)	55 (19.1)		15 (15.6)
	Refuse to answer or do not know	104 (27.1)	21 (21.9)	32 (33.7)	24 (25)	27 (27.8)	75 (26)		29 (30.2)

^a^RCT: randomized controlled trial.

^b^Group A: 25 Health Points per completed ecological momentary assessment survey.

^c^Group B: 25 Health Points per completed ecological momentary assessment survey + bonus Health Points available.

^d^Group C: 50 Health Points per completed ecological momentary assessment survey.

^e^Group D: 50 Health Points per completed ecological momentary assessment survey + bonus Health Points available.

### Aim 1: Reward RCT

The first aim was to evaluate whether changing the reward structure for completing EMA surveys would lead to an increase in the response rate.

The average response rates for the 4 reward groups at burst 1 and burst 2 are presented in Table 3. The response rate at burst 2 increased for all groups (compared with that at burst 1). However, the differences in the burst 2 response rate between the groups were not significant (Table 3).

**Table 3:** Response rate (%) by reward structure for the 4 intervention groups.

Group	Burst 1, mean (SD)	Burst 2, mean (SD)	*F* test (*df*), unadjusted^a^	*P* value	*F* test (*df*), adjusted^b^	*P* value
A^c,d^	24.42 (29.71)	50.56 (33.61)	1.75 (3, 380)	.16	1.38 (3, 376)	.25
B^e^	24.34 (31.13)	41.44 (35.98)	N/A^f^	N/A	N/A	N/A
C^d,g^	27.08 (30.00)	43.85 (34.16)	N/A	N/A	N/A	N/A
D^h^	27.24 (29.96)	50.49 (34.16)	N/A	N/A	N/A	N/A

^a^ANOVA.

^b^Analysis of covariance, adjusted for burst 1 response rate (baseline).

^c^Group A: 25 Health Points per completed ecological momentary assessment survey.

^d^Participants in group A or group C could receive either the 50% or 80% completion bonus, not both.

^e^Group B: 25 Health Points per completed ecological momentary assessment survey + bonus Health Points available.

^f^N/A: not applicable.

^g^Group C: 50 Health Points per completed ecological momentary assessment survey.

^h^Group D: 50 Health Points per completed ecological momentary assessment survey + bonus Health Points available.

### Aim 2: Schedule Length RCT

The second aim was to evaluate whether implementing a shortened 7-day EMA schedule would improve the response rate.

The average response rates for the 2 schedule length groups are shown in Table 4.

On average, participants in the 14-day group (control) completed 48.3% (SD 33.2%) of EMA surveys at burst 3 compared with 38.5% (SD 33.4%) in the 7-day group (intervention); this difference was significant (*F*_1,382_=6.23; *P*=.01) and remained so after adjusting for the response rate at burst 1 (baseline; *F*_1,380_=4.63; *P*=.03; Table 4).

**Table 4:** Response rate (%) by schedule length groups.

Group	Burst 1, mean (SD)	Burst 3, mean (SD)	*F* test (*df*), unadjusted^a^	*P* value	*F* test (*df*), adjusted^b^	*P* value
7-d schedule (intervention)	24.09 (29.18)	38.52 (33.44)	6.23 (1, 382)	.01	4.63 (1, 380)	.03
14-d schedule (control)	30.85 (32.39)	48.34 (33.17)	N/A^c^	N/A	N/A	N/A

^a^ANOVA.

^b^Analysis of covariance, adjusted for burst 1 response rate (baseline).

^c^N/A: not applicable.

### Secondary Aim 3: Temporal Trends in Response Rate

At baseline (ie, burst 1), the average response rate per participant was 25.8% (SD 30.1%). At burst 2, the average response rate across groups was 46.6% (SD 34.6%) and 41% (SD 33.6%) at burst 3, and this difference was statistically significant (*F*_2,766_*=*93.83; *P*<.001). Pairwise comparisons indicated that the difference between burst 1 and burst 2 and burst 1 and burst 3 were both significant (*P*<.001), whereas the difference between burst 2 and burst 3 was not significant (*P*=.05).

Subgroup analysis with control group participants was used to compare the overall response rates across the 3 bursts of EMA surveys following changes to the EMA implementation protocol. Table 5 shows the average response rate for all participants at burst 1 (N=384) and for participants allocated to the control conditions at burst 2 (group A, n=96) and burst 3 (14-d schedule group, n=96; note that 24 participants contributed data to all 3 bursts). The average response rate was higher at burst 2 (50.6%, SD 33.6%) and burst 3 (48.3%, SD 33.2%) than at burst 1 (25.8%, SD 30.1%), but the difference was not statistically significant (*F*_4,215_*=*0.72; *P*=.58).

Multimedia Appendix 3 shows details of how the response rate varied across each burst of EMA surveys.

**Table 5:** Temporal trends in response rate.

		Burst 1, mean (SD)^a^	Burst 2, mean (SD)^b^	Burst 3, mean (SD)^c^	*F* test (*df*)	*P* value
**Temporal trends, all participants**						
	Response rate	25.78 (30.12)	46.61 (34.58)	40.97 (33.60)	93.83 (2, 766)	<.001
**Temporal trends, subgroup analysis of control group participants at each burst^d^**						
	Response rate	25.78 (30.12)	50.56 (33.61)	48.34 (33.17)	0.723 (4, 215)	.58

^a^Includes all 384 participants from burst 1.

^b^Includes 96 participants who were allocated to group A (control) at burst 2.

^c^Includes 96 participants who were allocated to the four 14-day schedule groups (control) at burst 3.

^d^A total of 24 participants contributed data to all 3 bursts.

## Discussion

### Principal Findings

This study experimentally evaluated the effect of rewards and schedule length on EMA response rates within the context of an ongoing study implementing repeated bursts of EMA surveys. Reducing the number of days of EMA surveys led to a significantly lower response rate. However, changing the available rewards did not significantly change the response rate. Overall, for all groups the response rate was lowest at baseline (burst 1) as compared with the subsequent bursts of EMAs and the difference was statistically significant. However, our subgroup analysis that was intended to further explore whether this was because of changes in how participants were notified of the relevant burst of EMA surveys found no significant difference in the response rate at each burst.

The response rate to burst 1 was very low, prompting this study, and increased substantially in burst 2 and burst 3 in all groups. It is possible that the initial low response rate was because the upcoming EMA burst was not well communicated to the participants. Future bursts included more frequent communication delivered directly to all participants (via SMS text messages and push notifications). These simple changes may have contributed to the increased response rate. However, we did not experimentally evaluate the effects of these communication changes. Our secondary analysis partially supported this finding, as there were significant differences between the response rate at each burst. However, in the subgroup analysis of the control group participants, the differences were no longer significant.

### Findings in Context

The finding that neither offering greater reward amounts nor reducing the schedule length led to an increase in the response rate is broadly consistent with systematic reviews of factors associated with EMA compliance [8,32]. However, these reviews highlight the inconsistencies in how response rates are reported (eg, of studies in nonclinical populations, only 22% reported average response rate/person [8]), which makes direct comparisons challenging. Greater uptake of EMA study reporting guidelines [20,38] would be useful in this regard. Our study extends the currently available evidence by providing an experimental evaluation of the role of rewards and schedule length.

Rewards were selected as the first intervention target as the rewards available in burst 1 were low compared with other studies; participants could receive a total of approximately US $5 for completing all the EMA surveys. In other studies with a similar number of EMA days (between 10 and 14 d) and surveys (between 35 and 50 surveys), the lowest incentive was approximately US $25 [14]. In burst 2, the total available rewards increased for some groups (up to US $15) but remained lower than comparable studies and there was only a US $10 difference between the lowest and highest value reward group. In our study, very small rewards were directly tied to the completion of each individual EMA survey (ie, 25-50 HP, approximately US $0.12-US $0.24/completed EMA survey). In the context of Singapore, small incentives (in the form of HP and supermarket vouchers) have been used to promote compliance to interventions [45,46], although in these instances, the relationship between intervention compliance and the incentives available may have been clearer to participants. In contrast, Health@NUS participants have a range of different study requirements that are tied to different incentives; over the course of the 2-year study, participants can receive up to about US $313 (plus keep their study Fitbit). In addition, participants were likely aware that completing the EMA surveys was optional (but strongly encouraged). Taken together, immediate rewards may have seemed small for all reward groups, and cost-benefit reasoning may have resulted in a decision to not complete the EMA surveys [47].

In our study, contrary to our expectations, the burst 2 response rate was significantly higher in the 14-day schedule group than in the shorter 7-day schedule group. It seems intuitive that fewer days of EMA surveys would be less intrusive and therefore preferable to participants, particularly in the context of repeated bursts of EMA surveys. However, our findings indicate the opposite. More research is needed in this area as systematic reviews currently report inconsistent relationships between EMA schedule length and response rate [8,32].

Although the average response rate in EMA studies has been reported to be >70% [11,38], these studies implement a single burst of surveys rather than repeated bursts. Our results compare favorably with those of 2 other studies that have used burst designs. In the SPARC study [48], 4 bursts of EMA surveys (8 surveys/d for 4 d) were implemented over a 7-month period (September, October, February, and March) and reported an average per participant response rate of 41% across the 4 bursts [49]. Similarly, Howland et al [50] asked participants to complete a 30-day daily diary annually for 4 years. The retention rate across the 4 years was reported as 73%; however, this was after participants who did not meet the minimum reporting threshold of 15 days per year (out of 30 days) were excluded from the study. It is important to note that these 2 studies [48,50] only implement repeated EMA and traditional questionnaires (no continuous or in-person assessments). As such, in these studies, participants would likely have specifically signed up to an EMA study, as compared with Health@NUS participants who have numerous other elements of data collection to fulfill, with the EMA secondary to this and optional. This highlights potential challenges with repeatedly administering EMA (in or outside the context of a larger study) and that researchers should carefully consider the likelihood of missed EMA surveys (and missing data) when using burst designs [20,21]. However, further research is required to confirm our findings. Two recent reviews of EMA compliance found no evidence of a significant relationship between schedule length and compliance [8,32], although these data were obtained from observational rather than experimental studies. There are few experimental studies of study design features to minimize missing EMA data [33] and few longitudinal EMA studies [23-25].

### Strengths and Limitations

The strengths and limitations of this study should be considered. Our RCTs were embedded within an ongoing cohort study that required participants to fulfill several mandatory requirements (eg, minimum Fitbit wear time and food diary logging/mo), whereas completing the EMA surveys was optional but highly encouraged, and participants may have decided to opt out of this study component. Furthermore, although our sample size was larger than that of many other EMA studies [11,38], no a priori sample size was calculated and instead all participants who enrolled during the first waves of recruitment (October 2020 to March 2021) were included. Our study is one of the first to experimentally evaluate the impact of EMA protocol features on overall response rates, and we purposefully chose protocol features that could be manipulated and evaluated within an RCT. However, as the first few bursts of EMA were organized and scheduled in advance, the research team had to rapidly analyze the previous bursts’ response rate data and select a suitable intervention strategy for the upcoming burst. Given more time, we may have selected alternative variables to manipulate, such as time-varying factors (eg, time of day or weekend vs weekday [51]); number of EMA surveys per day (eg, our varied number of EMA surveys/d vs a consistent number); or whether the response rate can be predicted based on the question type (eg, Likert scale or multiple choice) or content (eg, dietary intake or stress). Future studies should explore the role of time-varying factors, the number of EMA surveys, and the type and content of questions on the overall response rate.

Our pragmatic approach also meant that our secondary aim, to explore temporal changes in the response rate, was exploratory in nature. Future studies specifically designed to experimentally evaluate the effect of altering the announcement and communication strategy for each EMA burst (ie, the number of emails, SMS text messages, and push notifications that were sent to provide details of what to expect in the upcoming burst of EMA surveys) are needed. Studies evaluating the effects of other temporal variables such as holidays and key periods in the academic calendar (eg, exams) are also needed. As is typical of behavioral research, it was not possible to blind participants to their intervention condition, and we also cannot comment on whether participants received all of the EMA surveys that were sent. Furthermore, in our study, the number of EMA surveys per day varied (3-6/d), which may have lowered the response rate as participants did not know when to expect a survey. Finally, our sample consists of university students who may be especially motivated to engage in health research and therefore may not be representative of the broader population of young adults. The extent to which our findings generalize beyond this group is unclear. However, as young adults are a key population studied in EMA studies [20,32,52-55], our findings are likely to be of considerable interest to the field.

### Conclusions

This study is one of the first to experimentally evaluate the effect of incentives and schedule length on EMA response rates. It is also the first study to consider factors related to response in the context of an ongoing prospective cohort study administering repeated bursts of EMA over a 2-year period. By embedding RCTs within an ongoing study, it was possible to rapidly implement and evaluate whether altering the implementation strategy, incentives, or schedule length would increase the response rate. Our study therefore contributes to a small but growing body of literature on how to implement EMA. This knowledge is essential for collecting high-quality EMA data, which has a flow-on effect to the quality of conclusions that can be drawn from these data.

## Appendix

Multimedia Appendix 1 Overview of the 14- and 7-day ecological momentary assessment schedules including number of questions sent per ecological momentary assessment survey.

Multimedia Appendix 2 Constructs assessed via Ecological Momentary Assessment surveys—example questions and response options.

Multimedia Appendix 3 Per day response rate for each burst of Ecological Momentary Assessment surveys.

Multimedia Appendix 4 CONSORT-ROUTINE (Consolidated Standards of Reporting Trials extension for the reporting of randomised controlled trials conducted using cohorts and routinely collected data) checklist.

Multimedia Appendix 5 CONSORT-EHEALTH checklist.

## Abbreviations

CONSORT: Consolidated Standards of Reporting Trials
EMA: ecological momentary assessment
HP: Health Points
NUS: National University of Singapore
RCT: randomized controlled trial

## Data Availability

Deidentified data that support the findings of this study may be available from the corresponding author (SME) upon reasonable request.
