# Supplementary figures and images for: Evaluating the Effects of Rewards and Schedule Length on Response Rates to Ecological Momentary Assessment Surveys: Randomized Controlled Trials
Source: J Med Internet Res. 2023 Oct 19;25:e45764. doi: 10.2196/45764 (PMC10623229; doi:10.2196/45764)

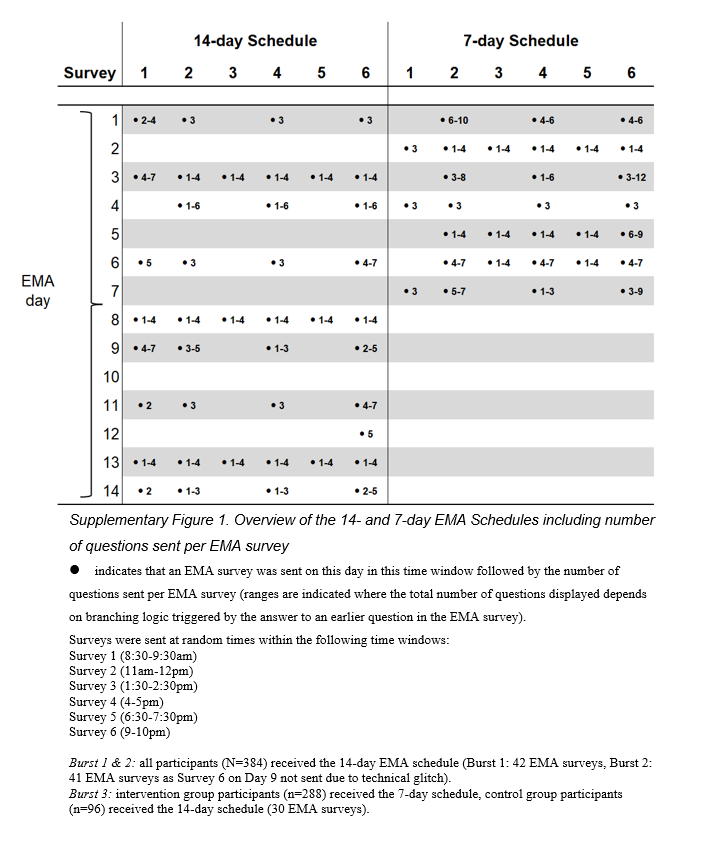

Supplement: Multimedia Appendix 1 [file jmir_v25i1e45764_app1.png]

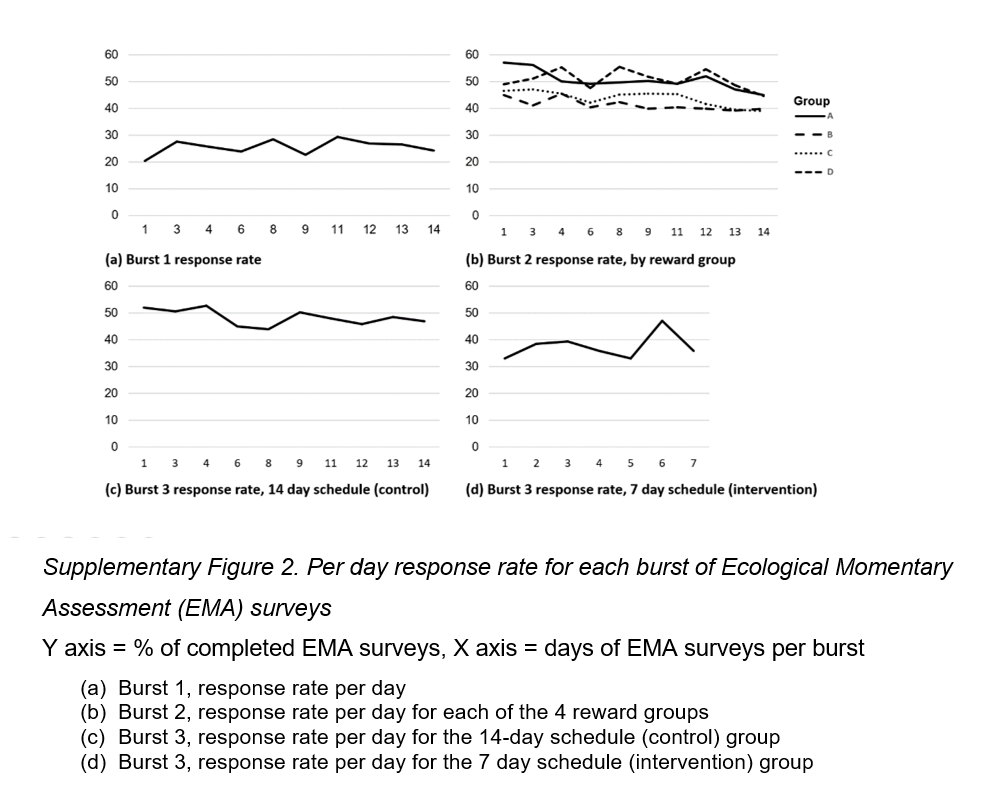

Supplement: Multimedia Appendix 3 [file jmir_v25i1e45764_app3.png]
