# Supplementary material for: Evaluating the Effects of Rewards and Schedule Length on Response Rates to Ecological Momentary Assessment Surveys: Randomized Controlled Trials
Source: J Med Internet Res. 2023 Oct 19;25:e45764. doi: 10.2196/45764 (PMC10623229; doi:10.2196/45764)
Supplement: Multimedia Appendix 2 [file jmir_v25i1e45764_app2.docx]

| Supplementary Table 1. Constructs assessed via Ecological Momentary Assessment (EMA) surveys – example questions and response options | |
| --- | --- |
| **Construct** | **Example EMA question and response options** |
| Sleep | How would you rate your overall sleep quality last night?  Likert scale: 0=very bad, 6=very good |
| Screen time | Did you use a screen over the past 3 hours?  Categorical: yes, no |
| Diet | Did you eat a meal, snack or drink (other than water) over the past 3 hours?  Categorical: meal, snack/desserts, drink |
| Physical activity | Have you done any exercise today?  Categorical: yes, no |
| Stress | How stressed do you feel right now?  Likert scale: 0=very bad, 6=very good |
| Happiness | How happy do you feel right now?  Likert scale: 0=very bad, 6=very good |
| Fatigue | How tired do you feel right now?  Likert scale: 0=very bad, 6=very good |
